# How Do Family Caregivers’ Values Influence Pain Management for Older Adults with Dementia?

**DOI:** 10.1093/geroni/igab046.3402

**Published:** 2021-12-17

**Authors:** Hui Zhao, Pamela Kulbok, Ishan Williams, Carol Manning, Rafael Romo

**Affiliations:** 1 James Madison University, Harrisonburg, Virginia, United States; 2 University of Virginia, Charlottesville, Virginia, United States; 3 Dominican University of California, San Rafael, California, United States

## Abstract

Professional caregivers rely on formal training when managing pain among patients with dementia, but family caregivers (FCGs) lack this foundation. Instead, FCGs use informal sources that may reflect a values-driven decision-making process. Few studies have examined how FGCs’ personal values impact pain management for dementia patients. We sought to examine the influence of personal values on pain management among FCGs for community-dwelling older adults with dementia using qualitative descriptive methods. Twenty-five adult FCGs, aged from 29 to 95, were recruited in central Virginia. Participants were predominantly white, married, female, and high school graduates. We conducted semi-structured interviews that were audio recorded and analyzed using constant comparative analysis. Four themes emerged: 1) Priority for pain management: when quality of life is valued over other factors (i.e., length of life), priorities focused on no pain, leading to better pain management; 2) Moral perspectives: negative views toward drugs, especially opioids, led to less use and greater report of pain; 3) Beliefs about alternative therapy: negative views led to less likely use of non-traditional approaches and reports of more pain, and 4) Personal experience of pain: past personal experiences of pain (negative or positive) influenced the priority placed on pain management and the FCG’s ability to provide effective pain management. The diverse views held by FCGs demonstrate a value-based process and suggest a modifiable factor in pain management. Helping FCGs reflect biases while reinforcing values that improve pain management would lead to improve pain and quality of life for older adults with dementia.

